# Developing public health competency statements and frameworks: a scoping review and thematic analysis of approaches

**DOI:** 10.1186/s12889-023-17182-6

**Published:** 2023-11-13

**Authors:** Melissa MacKay, Caitlin Ford, Lauren E. Grant, Andrew Papadopoulos, Jennifer E. McWhirter

**Affiliations:** Department of Population Medicine, University of Guelph, Guelph, ON N1G 2W1 Canada

**Keywords:** Public health, Competencies, Competency frameworks, Knowledge, Skills, Behaviours, Values

## Abstract

Competencies ensure public health students and professionals have the necessary knowledge, skills, values, and behaviours to do their jobs effectively. Public health is a dynamic and complex field requiring robust competency statements and frameworks that are regularly renewed. Many countries have public health competencies, but there has been no evidence synthesis on how these are developed. Our research aim was to synthesize the extent and nature of the literature on approaches and best practices for competencies statement and framework development in the context of public health, including identifying the relevant literature on approaches for developing competency statements and frameworks for public health students and professionals using a scoping review; and, synthesizing and describing approaches and best practices for developing public health competency statements and frameworks using a thematic analysis of the literature identified by the scoping review. We conducted a scoping review and thematic analysis of the academic and grey literature to synthesize and describe approaches and best practices for developing public health competency statements and frameworks. A systematic search of six databases uncovered 13 articles for inclusion. To scope the literature, articles were assessed for characteristics including study aim, design, methods, key results, gaps, and future research recommendations. Most included articles were peer-reviewed journal articles, used qualitative or mixed method design, and were focused on general, rather than specialist, public health practitioners. Thematic analysis resulted in the generation of six analytical themes that describe the multi-method approaches utilized in developing competency statements and frameworks including literature reviews, expert consultation, and consensus-building. There was variability in the transparency of competency framework development, with challenges balancing foundational and discipline-specific competencies. Governance, and intersectoral and interdisciplinary competency, are needed to address complex public health issues. Understanding approaches and best practices for competency statement and framework development will support future evidence-informed iterations of public health competencies.

**Supplementary Information:**

The online version contains supplementary material available at 10.1186/s12889-023-17182-6.

## Background

Competencies are defined as the knowledge, skills, values, and behaviours required to perform well within a profession and an organization [1]. Knowledge can be acquired through education and experience [1]. Skills result from the repeated application of knowledge, and behaviours reflect how individuals perform in various contexts [1]. Attitudes and values form the context in which competencies are practiced and are the beliefs that motivate people to act in different ways [2]. Competencies required for professional practice depend on many factors including an individual’s position within an organization and an organization’s needs [1, 3]. Within education, curriculum and pedagogical approaches provide students with opportunities to advance their knowledge and skills through didactic and experiential training [4, 5]. For both professional development and education, defining the competencies needed and creating development opportunities explicitly linked to them is essential.

There are various conceptualizations of competencies, including behavioural, performance continuum, and integrated or holistic approaches. Each conceptualization has strengths and weaknesses based on the complexity and interconnectedness of the approach, its focus on tasks or job performance, and how competencies are developed within individuals during their careers [6]. The holistic approach to competence conveys the interrelationship of knowledge, skills, behaviours, and attitudes/values [6, 7]. Frameworks and approaches to competencies are context-dependent and developmental, where there is progressive interaction between an individual’s tasks, abilities, and the systems and environments in which they perform [6]. Thus, establishing an integrated competency framework for any discipline or sector, including public health, provides clarity and professional standards, promotes reflective practice, and requires a clear understanding of the interconnectedness of the attributes required.

Competency statements for public health ensure students and practitioners have the necessary knowledge, skills, behaviours, and values to effectively perform [2, 8]. This promotes standardization in the field and provides a benchmark for capable personnel, allowing for a consistent approach to organizational planning and performance measurement, which in turn improves the quality of public health programs and services [5]. Competency frameworks define a set of competency statements and support their implementation through describing public health practice expectations and informing workforce planning and development [9]. There are a number of frameworks for public health competencies around the world, including the Core Competencies for Public Health in Canada [2], the United States (US) Core Competencies for Public Health Professionals [8], the WHO-ASPHER Competency Framework for the Public Health Workforce in the European Region [10], the Public Health Competencies and Impact Variables for Low- and Middle-Income Countries [11], the Asia-Pacific Academic Consortium for Public Health Competency Framework [12], and the Core Competencies for Public Health: A Regional Framework for the Americas [13].Although each framework consists of different conceptualizations of competencies for different countries and sociopolitical regions, they are all based on a need for a tool that facilitates development of a knowledgeable and skilled public health workforce able to effectively address complex public health challenges [4].

Current public health challenges include multiple crises including climate change [14], mental health [15], and opioid use disorder [16], which overlap many sectors and disciplines. The COVID-19 pandemic exacerbated existing global health issues including health inequities, mental health, social isolation, and addictions [17, 18]. Additionally, declining trust in public health resulted from ineffective crisis communication and management [19], and a complex information ecosystem where mis/disinformation is widely circulating [20]. More than ever, public health needs competency frameworks that reflect the current multifaceted and overlapping public health challenges to ensure the workforce is adequately equipped to address them.

Core competency frameworks in public health are usually developed through collaboration with a range of public health experts and professionals. Public health competency frameworks are developed at various levels including the country level, organizational level, and discipline-specific public health practice to support workforce development planning, professional development, and improved public health action. For example, within Canada, the Core Competencies for Public Health were released in 2008 by the Public Health Agency of Canada in collaboration and consultation with public health practitioners, government, and experts [2]. Similarly in the US, the Core Competencies for Public Health Professionals are developed and updated through extensive consultation and revision with the public health and population health sectors across the country [21]. This work also occurs on an organizational level within academia to develop programs and curriculum matched to public health competency frameworks [22], as well as for specific disciplines within public health such as implementation science [5], public health preparedness and response [23], and environmental public health [24].

The field of public health is dynamic and complex; thus, competency frameworks must be regularly updated to remain relevant. Approaches for developing public health competency statements and frameworks must be optimized and mobilized to ensure they are relevant and have impact within public health [25]. Renewal of public health competency frameworks varies globally, with some countries regularly updating their competencies, such as the US (considers renewal three years after the prior release), while others, such as Canada, do not currently have a systematic approach to updating competencies, though there are calls for renewal and efforts are underway to revisit this. Identifying and describing approaches for the development of public health competency statements and frameworks will support future evidence-informed iterations, but no previous evidence synthesis has been conducted.

To address this gap, our goal is to synthesize the extent and nature of the literature on approaches and best practices for public health competencies statement and framework development. The objectives are:

1. Identify relevant literature on approaches for developing competency statements and frameworks for public health students and professionals using a scoping review; and,
2. Synthesize and describe approaches and best practices for developing public health competency statements and frameworks using a thematic analysis of the literature identified in the scoping review.

## Methods

### Review approach and team

The scoping review methods were based on the framework outlined by Arskey and O’Malley [26] and updated by Levac et al. [27]. A research team with expertise in the subject matter and methods was established to develop and guide the scoping review protocol and thematic analysis [26, 27]. A specialist research librarian with expertise in public health and research synthesis was consulted for the scoping review protocol. This scoping review is reported in accordance with the PRISMA statement for scoping reviews [28].

### Review scope

Articles were included if they were published in English. There were no geographic or date restrictions. Peer-reviewed journal articles with qualitative, quantitative, and/or mixed methods study designs, dissertations, and grey literature were included. Literature was included when it focused on developing competency statements and frameworks for public health students and practitioners. Competency development in other disciplines (e.g., medicine, dentistry, etc.) was excluded unless it was explicitly public health focused (e.g., public health nursing).

### Search strategy

In collaboration with the research team and a specialist research librarian, the search strategy was developed by exploring the relevant literature for keywords and controlled vocabulary. Originally, our review intended to focus on communication-related competencies for public health specifically, so controlled vocabulary and keywords were included to reflect this focus. During the screening process, it became apparent there was not enough literature focused on public health communication. The search strategy was expanded to focus across all competencies relevant to public health, including communication.

The search strategy was tested in Ovid via MEDLINE and refined to ensure the results were relevant to the review scope. The search strategy was then translated for use in other databases by modifying syntax, as needed. The final search was carried out on November 24, 2022, in the following databases: Ovid via MEDLINE (Table 1), PsycINFO, Web of Science, Communication and Mass Media Complete, ERIC, and CAB Direct. These databases were selected to provide a comprehensive coverage of public health, education, and communication sources.

**Table 1:** Controlled Vocabulary and Keywords Used in Ovid via MEDLINE

MeSH Term(s)	Keywords
Health Communication	“health communication” OR communication OR “health information” OR “health informatics” OR dissemination OR “knowledge translation” OR “behaviour change” OR awareness OR attitude* OR knowledge OR “social marketing” OR “social media” OR “communication channel*” OR “health literacy” OR “mass media” OR “cultural competency” OR “online health communication” OR “risk perception” OR “risk communication” OR “crisis communication” or “health ethics”
Public Health Practice	“public health” OR “public health practice” OR “public health practitioner*” OR “public health workforce” OR “public health capacity” OR “public health capabilit*” OR “evidence-based public health practice” OR “public health impact” OR “practitioner knowledge”
Students, Public Health Education, Public Health Professional Professional Education Curriculum	pedagogy OR education OR learning OR teaching OR curriculum OR curricula OR “transmission of knowledge” OR “professional development” OR “Master of Public Health” OR “public health training” OR “public health education” OR “training program*” OR “public health graduate training” OR “public health graduate education” OR “competency-based curricul*” OR “competency-based assessment*” or competency or understanding or qualification* or categor* or “competency-based curricul*” or “competency-based assessment?“ or skill* or “know how” or “competency adj5 belief*” or “competency adj5 value*” or “competency adj5 attitude*”

To supplement the database search, we hand-searched the following journals: *Journal of Public Health Management and Practice, American Journal of Public Health, Journal of Health Communication, Health Promotion Practice*, and *Health Communication*. The journals were identified by examining the most frequently cited journals in the database search results. Additional peer-reviewed articles were also identified during the grey literature search. Grey literature was searched using Google through appropriate combinations of concepts (e.g., “core competencies” AND “public health”) and pages 1–10 were screened for relevant results.

### Relevance screening

The results of the search were imported into DistillerSR review software [29] to facilitate screening by independent reviewers and track all steps. Deduplication of results occurred in DistillerSR after all relevant citations were imported from Mendeley [30]. Title and abstract, and full-text screening were also conducted in DistillerSR. Reviewers (MM and CF) pilot tested ten random articles to ensure understanding of the inclusion criteria. Two independent reviewers (MM and CF) screened the title and abstract of each article using a screening form and any conflicts were resolved through discussion. Kappa was 0.81 for title and abstract screening, indicating high agreement [31].

The full text of articles deemed potentially relevant during the title and abstract screening were obtained and screened independently by the same two reviewers. Steps were taken to obtain the full text of all articles including searching within the University of Guelph Library, using Google and Google Scholar, and contacting the author directly through ResearchGate or their publicly available institutional email. A screening form was developed in collaboration with the research team and pre-tested before implementation. The form assessed each article’s eligibility for inclusion in the review by the following criteria: literature type, language, population, and measurement, evaluation, or detailed report of competency statement or framework development in public health students and/or the workforce. After completion of full-text screening, Kappa was 0.80, indicating high agreement [31]. Conflicts were resolved through discussion to the point of reaching agreement for inclusion or exclusion in the review.

### Data extraction

Two researchers conducted data extraction for the included articles (MM and CF). Each researcher acted as the primary data extractor for half of the dataset and validated the other half as the secondary data extractor. Key information was extracted using an Excel form developed in collaboration with the research team [32]. The following information was extracted from each included article: title, author(s), year, article type, country of origin, study design, study aim, methods, theories or frameworks included, institutions involved in developing competency statements, existing competency frameworks included, focus of competency framework (e.g., general or discipline-specific), process used to develop competency statements or framework, level of competency development focus (e.g., nation-wide, organizational, etc.), target population (e.g., graduate student, general public health practitioner), transparency of the process, lessons learned, implementing and adoption of competency statements or framework, evaluation of the process, bias identified, and future research directions.

The results of the data extraction were thematically analyzed following the method outlined by Arskey and O’Malley [26] and updated by Levac et al. [27]. First, the research team developed key areas to capture results for data extraction to answer objective 2, including transparency of methods and results, evaluation of the process, lessons learned, and implementation/adoption of the competencies. One researcher (MM) coded the extracted data line-by-line, as well as revisited the full text articles, to develop an initial thematic framework that described approaches and best practices for developing competency frameworks in public health. Codes were inductively created based on the key areas of data extraction outlined above and refined into larger concepts where data overlapped to generate initial themes. Significant outliers were captured in the coding and incorporated into themes where appropriate. Approaches for developing competency statements and frameworks described any methods undertaken and/or key recommendations for generating competency statements for public health that outline the values, knowledge, skills, and behaviours needed to effectively perform in various public health roles. Best practices describe areas of significant overlap in the data that reported successful methods or recommendations for developing, implementing, and/or evaluating competency statements and frameworks for public health. The research team collaboratively discussed and refined the themes to provide perspective and triangulation of results, ultimately developing six analytical themes. The research team also identified any areas of ambiguity during discussion and/or revisions and MM revisited the extracted and coded data, as well as full text articles, to add detail. The research team then collaboratively discussed, refined, and finalized the analytical thematic framework.

## Results

### Search and selection of articles

**Fig. 1:**
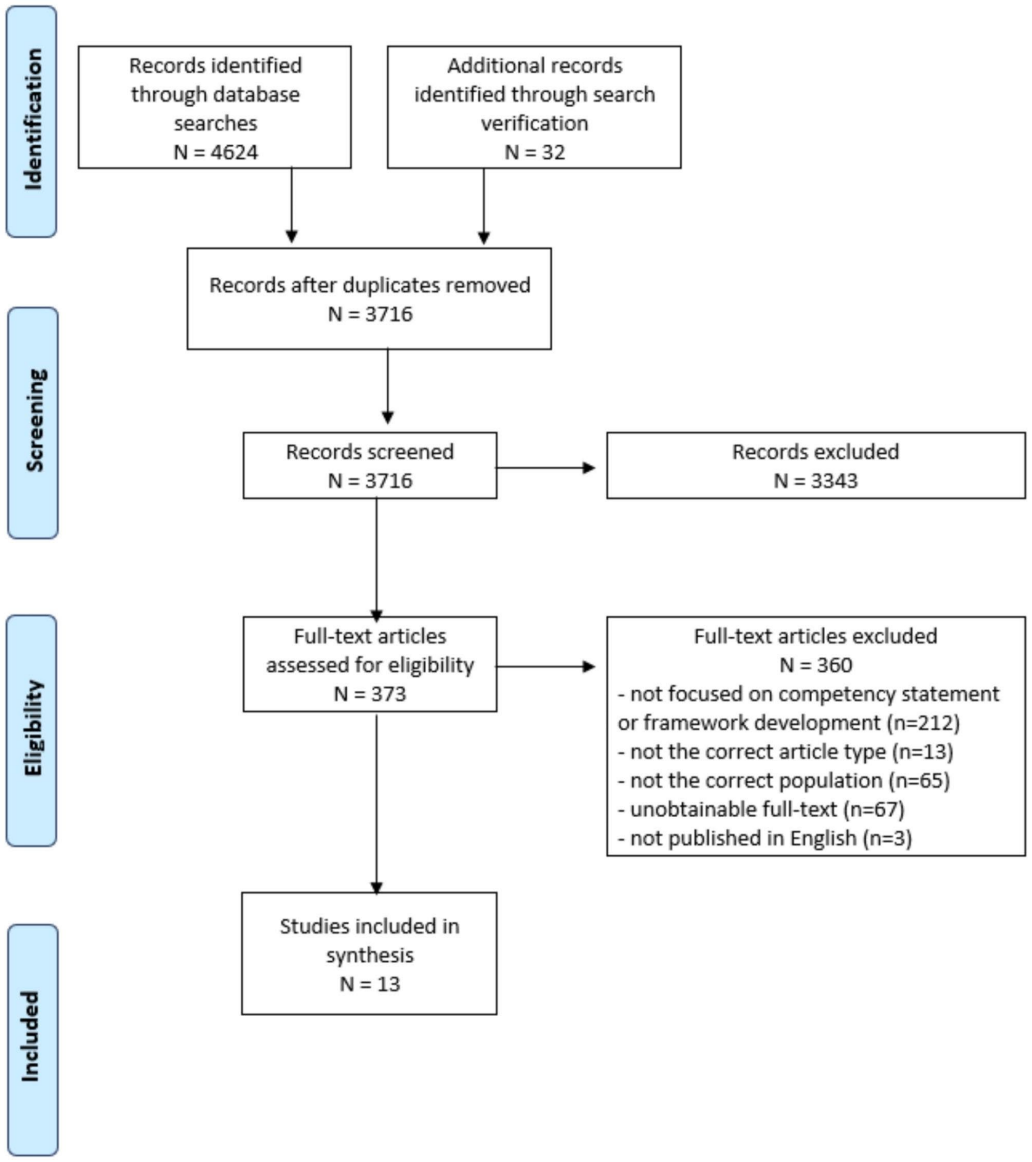
PRISMA flow diagram of scoping review process

A total of 3,716 articles were screened at the title and abstract stage following deduplication. Next, 373 full-text articles were reviewed for relevance, with 13 studies identified for inclusion. Figure 1 depicts the PRISMA flow diagram of the article screening and inclusion process.

### Characterization of Articles included

Most included articles were peer-reviewed journal articles, used qualitative or mixed method design, and were targeted towards generalist public health practitioners (Table 2). See Supplementary Table 1 for the data extraction results.

**Table 2:** Summary Characteristics of Articles (n = 13) on Public Health Competency Statement and Framework Development

Characteristic			n	%^+^
*Document Type*				
Journal Article ^23–25, 27, 29–35^			10	77
Grey Literature ^26, 30^			2	15
Conference Report ^28^			1	8
*Study Design*				
Qualitative ^23, 26–28,31, 35^			6	46
Mixed Methods ^24–25, 29, 33–34^			5	38
Quantitative ^32^			1	8
N/A (Grey Literature) ^26, 30^			2	15
*Region or Country of Origin*				
U.S.A ^23–25, 27, 30, 32^			6	46
Canada ^34, 26^			2	15
United Kingdom ^31, 35^			2	15
China ^33^			1	8
Ireland ^28^			1	8
India ^29^			1	8
Target Population for Competency Framework*				
General Practitioner ^23–24, 26, 30–31, 35^			6	46
MPH Students ^25, 29^			2	15
Epidemiologists ^34^			1	8
Public Health Physicians ^33^			1	8
Graduate Students ^32^			1	8
Health Promoters ^28^			1	8
Public Health Leaders ^29^			1	8
Theories and Frameworks*				
Benner Model of Expert Practice ^27^			1	8
Dreyfus Model ^27^			1	8
Estimate-Talk-Feedback-Estimate ^29^			1	8
Blooms Taxonomy of Educational Objectives ^24^			1	8
Knowledge Skills Abilities Model ^32^			1	8
Institutions Involved in Competency Development*				
Academia ^23–25, 27–34^			11	85
Government ^28–29^			3	23
Public Health ^29, 35^			2	15
Professional Association/Organization ^26, 28^			2	15
Existing Competency Frameworks Used*				
UK Public Health Skills and Knowledge Framework ^29, 31, 33, 25^			4	31
Core Competencies for Public Health Professionals ^25, 29, 33^			3	23
Core Competencies for Public Health in Canada ^26, 29, 33^			3	23
CDC Bioterrorism and Emergency Readiness ^27^			1	8
Public Health Association of Australia ^33^			1	8
Emergency Preparedness Core Competencies for Public Health Workers ^23^			1	8
Informatics Competencies for Public Health Professionals ^23^			1	8
Core Competencies for Public Health: A Regional Framework for the Americas ^29^			1	8
Public Health Association of New Zealand ^29^			1	8
*Focus of Competency Framework*				
General ^25, 29–31, 33, 35^			6	46
Epidemiology ^23, 34^			2	15
Emergency Preparedness ^27^			1	8
Health Literacy ^24^			1	8
Health Communication ^32^			1	8
Health Promotion ^28^			1	8
Indigenous Public Health ^26^			1	8
Methods Used to Develop Competency Framework*				
Survey ^23, 25, 27, 32–34^			6	46
Literature Review ^24–25, 27, 29, 32^			5	28
Expert Consultation ^25–26, 33, 35^			4	31
Interviews ^29, 31, 33–34^			4	31
Modified Delphi ^24, 27, 29, 34^			4	31
Conference ^28^			1	8
Focus Groups ^27^			1	8
Environmental Scan ^25^			1	8
*Level of Competency Framework Focus*				
Country ^23–24, 26, 29–35^			10	77
Organizational ^27^			2	15
International ^25, 28^			2	15

*More than one area for characteristic per article was possible, thus may not total 100%

^+^ Rounded to the nearest whole number

^23–35^ Indicates which characteristic that corresponds to the included articles

### Thematic analysis

Six themes were generated to describe the approaches and best practices for developing competency statements and frameworks for public health. The themes related to the approaches include initial methods used, building consensus, and transparency in reporting. Themes related to best practices include governance and coordination and a multifaceted approach for addressing complex public health challenges. The theme describing developing foundational and discipline-specific competencies and those that apply to varying levels of expertise included approaches and best practices.

### Initial competency statements are developed using literature reviews and expert consultations

Literature reviews [33–37], expert consultation [38–41], or a combination of both [35, 36, 42, 43] were used to develop initial competency statements.

As a first step, a literature review was often completed to identify competencies, including for public health physicians [43], health communicators [42], global public health practitioners [35], and for health literacy for public health professionals [34]. Grey literature, including existing country-level public health competencies (e.g., USA, Europe, Canada, New Zealand, Spain), existing competency frameworks (e.g., Emergency Preparedness Core Competencies for Public Health Workers), textbooks, and public health education curricula was frequently included in literature reviews for competency list development [33, 35, 37, 39, 43]. Less frequently, expert consultation was used as a first step in developing competency statements, including for emergency preparedness and response [37], public health epidemiology [44], and for senior public health officials [39]. Additional methods for getting started included conducting an environmental scan, which was used to develop Indigenous public health core competencies [36].

As a second step, expert consultations took place using interviews and surveys to gather information relevant to competency framework development, including feedback on competency statements, contextual factors, and responsibilities of and gaps in the workforce, including at various levels of public health practice [42, 43] and within academia [35]. Exemplar practitioners who demonstrate the competencies within well-established practices can model the knowledge, skills, and abilities required within a particular context [40, 41]; however, expert consultations are not an appropriate first step when the area of practice is emerging and there is no history of exemplar practitioners [34, 40]. Representation is important for expert consultations: Indigenous peoples’ leadership in creating an Indigenous competency framework ensured their beliefs, values, and knowledge were central [36]. Context, including culture, geography, experience, and education must be considered when identifying evidence and theory for competency list development [37].

While some of the included literature identified potential challenges in gathering and synthesizing diverse expert opinions within their methods [34, 36, 39, 41], no insights on how these were addressed during the research were shared in the results or discussion. One exception was that participants of a consensus-building process with less expertise had some difficulty understanding more complex competencies [41]. The authors did not elaborate on how this may have impacted the results or how they addressed this issue during the research, other than recommending that further research in this area is needed.

### Public health competency statement development is consensus-driven with practitioners and researchers

Expert consultation and validation by experts and practitioners must be included to increase the impact of competency frameworks [34, 40–44]. Expert consultation that provides repeated assessment of the competency statements increases the validity of the results [43], convergence of findings [34, 39, 44], and comprehensiveness of the framework [41]. Modified Delphi technique was frequently used to facilitate discussion between experts and allow for successive feedback of the group opinion on competency statements [34, 37, 39, 40]. For example, a Delphi technique to build competencies for bioterrorism and emergency readiness in public health gathered experts from food safety, epidemiology, occupational health, and Indigenous health and included various levels of expertise and roles, including Directors, staff, and community leaders [37].

Other techniques to develop consensus on competency frameworks included focus groups [37, 41], workshops [44, 45], a survey [42, 43], interviews with expert practitioners [43], an advisory committee [33], and a conference with experts [38]. The size of the consensus-building expert group should reflect the uncertainty in the literature, resources available, and the topic area within public health [34]. For example, an expert panel for developing health literacy competencies aimed to include at least 20 individuals from across a network of schools of health professionals, educators, and experts in health literacy to attend an in-person two-day meeting to ensure a variety and breadth of perspectives included [34].

Purposive sampling of experts to gather various levels of experience and expertise within various public health disciplines was frequently used [34, 37, 41–44]. Participants were recruited via professional association listservs [34, 42, 44], relevant government and community-based organizations [37], and public health agencies [41]. In terms of number of participants, studies did not include any information on *a priori* sample sizes for quantitative methods, however, qualitative methods were guided by the achievement of data saturation [41, 44].

As part of the consensus-building process, surveys are often used to gather feedback on agreement [34, 37, 42, 44] and importance [34, 35, 37, 40, 42] of competencies. Likert scales were used, including a 4-point scale of importance ranging from 1 (very important) to 4 (not important) [34], a 5-point scale of importance ranging from 0 (not used at all) to 4 (essential) [42], and a 5-point agreement scale ranging from 1 (strongly disagree) to 5 (strongly agree) [44]. Competency statement acceptance, rejection, or clarification based on feedback through surveys were sometimes reported [34, 44].

### Transparency of consensus-building processes for developing competency frameworks varied

Reporting transparency regarding how consensus on competency frameworks was reached varied. Some studies were very detailed [34, 39, 41, 42] while others less so [35, 38, 45].

Studies often reported response rates and agreement for all competency items included [34, 35, 39, 44]. Statistical analysis, usually as associations between agreement ratings, and agreement levels for included competency statements, were also reported [39, 42–44]. Statistical analysis included a factor analysis to reduce and synthesize the overall number of behavioural traits identified for public health physicians [43], means and standard deviations to identify participant characteristics and the importance of the competence statements [42], and bivariate analyses to determine if the level of agreement differed by participant characteristics [44]. A pre-determined threshold of acceptance was used by Coleman et al. [34] to increase the validity of findings.

Overall results of the process to build consensus on a final competency framework were reported by some studies but details were often lacking on how agreement was assessed [33, 37].

### Competency frameworks varied across foundational and discipline-specific competencies and levels of expertise

Competency frameworks must balance comprehensiveness with being targeted to various disciplines and roles within public health [39–41, 45]. While specialist competency frameworks are necessary, a coherent and unifying public health competency framework is needed to provide structure, guidance, and a common understanding of public health more broadly [39–41, 45]. There is overlap between competency skills, knowledge, and behaviours within competency domains and between foundational and discipline-specific frameworks [25, 46], making it more difficult to assess agreement or importance during consensus-building [34, 44].

Foundational competencies to address systemic factors related to colonialism and incorporate non-Western knowledge and Indigenous governance structures in public health are also necessary [36]. For example, community health workers are excluded from needing to have all the core competencies in the Core Competencies for Public Health in Canada 1.0 release [36]. However, these public health practitioners play a central role in Indigenous health and lack of proficiency in all the competencies can significantly impact Ingenious health [36].

Discipline-specific competencies define the roles within public health and guide professional development and education within the specialty [39–44]. In discipline-specific areas, such as emergency preparedness and response, competency development must reflect all potential public health areas of action and interdisciplinary and intersectoral action [37, 40]. Working collaboratively across sectors, disciplines, and at different levels of service enhances the impact of public health action and must be reflected in competencies for public health in discipline specific frameworks [38], as well as foundational frameworks.

Coordination across levels of jurisdiction, as well as credentialing practitioners from novice to expert levels of competence, facilitates integration of competencies into practice [37]. A model for lifelong learning for bioterrorism and emergency management is used to credential practitioners at varying levels of expertise from novice to expert, which combines education, practical experience, and learning outcomes to assess the level of expertise [37]. Similarly, competencies for public health can be approached at various levels of expertise including basic, advanced, and expert [37, 39, 41]. For example, verb changes can be made to competency statements to make them more appropriate for varying levels of expertise (e.g., understand vs. analyze vs. create) [41]. Competency theory can be used in combination with a foundational approach to create competency frameworks for public health where statements are interpreted for different functions in public health (e.g., communication, leadership, policy) [41].

Recognition and integration of competence acquired through experience, professional development, and strategic development toward organizational goals must be recognized, along with those acquired through formal education [37, 41, 44] and different forms of knowledge, including Indigenous ways of knowing [36]. Bhandari et al. [39] also note the distinction between competencies for public health students -- those that are expected to be obtained by the end of the education program and organized around academic disciplines – and professional competencies which reflect the needs of the workforce. The two are intricately related as professional competencies, or those needed to effectively perform on the job, should guide and inform educational competencies [39].

### Governance ensures competency frameworks are current, integrated into practice, and connected to professional development

Funding to establish indicators or performance measures for competencies to ensure their integration into practice is needed [38, 45]. Professional development in the competency areas is necessary for integration into practice and requires dedicated, ongoing funding [38, 45]. Common methods of competency assessment should be a key area of governance and funded programming within professional development [41]. Quality assurance mechanisms for professional development are needed that reflect the local context and should be implemented by training organizations and institutions [38]. Ideally, an independent administrative body should be instituted to develop and implement standards and quality assurance mechanisms [38].

Competency lists must be revisited and revitalized on a consistent basis in partnership with various sectors such as healthcare and academia [38, 40–42]. Discipline-specific competency frameworks must be reviewed and updated more regularly compared to general frameworks [40]. Competency frameworks that have not been updated for five years or more should be used with caution, especially in curriculum design, health communication, and organizational planning [40]. Overall, renewal should be planned for as part of the overall initial development of any framework and should include a survey of current practices to understand strengths and areas of opportunity [40].

A comprehensive plan for communicating the results of developing competency frameworks ensures competencies are adopted by public health organizations [38] and the work is not duplicated [40]. Various formats for competency frameworks were used, including Tables  (33,34,39,42,43), lists [35, 37, 38, 41], visual models [36, 43, 45], and concept maps [44]. Only one study included any evaluation of the usefulness of the design and found simple and concise layouts were preferred [41].

Competency frameworks provide organizations and policymakers with a tool by which they can address their specific context and needs through workforce planning, including developing job descriptions, performance indicators, and professional development opportunities [39, 40]. New and revitalized frameworks should be mapped against job descriptions in public health to identify gaps [39]. Practitioners were apprehensive around competency frameworks being used for accreditation and performance evaluation [44]. Training programs should be designed and assessed to ensure they are able to develop the competencies that are outlined within frameworks [39–42]. Professional development for the current public health workforce must be integrated within the development and implementation of public health competency frameworks [34, 39, 41, 42, 44, 45].

### Values underpin and support competency frameworks to enable public health to address complex public health challenges

Values should be reflected in competency statements, although these are the most difficult to develop and measure [36, 38, 43]. A reflexive practice to identify the values and beliefs that guide public health practice would contribute to intercultural competency [36]. Values must include a commitment to health equity, social justice, intercultural competency, climate justice, and others that are rooted in the social ecological model of health and the social determinants of health [35, 36, 38, 42].

Although many frameworks highlight the importance of reducing health inequities (e.g., in the preamble), they varied in terms of the emphasis placed within competency statements on reducing health inequities and culturally appropriate approaches to public health [36, 39]. Competencies for providing culturally appropriate public health and healthcare for Indigenous peoples are vital and can contribute to overcoming misunderstanding, discrimination, and racism [36]. The explicit recognition of Indigenous peoples, colonialism and the impact on health, and related health inequities throughout the public health core competencies, including integration within definitions in the glossary and practice examples, is necessary [36].

Public health competencies are necessary to facilitate shared understanding of expectations and actions required to address complex public health issues that are intersectoral and interdisciplinary [38, 41, 45]. The ability to influence policy and develop partnerships can be negatively influenced by predominant culture and requires political will and in some cases governmental reform to ensure competencies to address complex public health issues are included in public health frameworks and practice [36, 38, 41, 43]. A flexible, action-oriented, and regulated public health workforce is needed to address serious and complex public health challenges, including climate change and health inequities [45]. Greater integration of competency frameworks with relevant legislation, programs, and guidelines for the specific jurisdiction, as well as within global health, to address complex public health issues is recommended [44].

## Discussion

Overall, thirteen articles were identified related to approaches for developing competency statements and frameworks for public health, just one of which focused on communication competence. Most of the included literature was original, peer-reviewed research using qualitative or mixed-methods approaches. The main target population for competency framework development was generalist public health practitioners and the process was largely driven by academia. Foundational competency frameworks for general public health practice at the country level were the most commonly developed in the included studies. A range of approaches, especially in combination, were taken for developing competency statements frameworks, with consensus-building through modified Delphi techniques, expert consultation, and surveys commonly used. Below, we summarize the six themes described in the results and discuss these approaches and their importance for developing public health competency statements and frameworks (Table 3). In Table 3, the content within the Actions to Achieve Recommendation column summarizes key aspects of the results and discussion that relate to the success and impact of the approaches and best practices for developing competency statements and frameworks identified. We compare and contrast our findings to similar literature in veterinary medicine and healthcare to put the recommendations into a wider context and body of knowledge.

**Table 3:** Summary of approaches and best practices for developing evidenced-based, impactful public health competency statements and frameworks

Recommendation	Actions to Achieve Recommendation
Evidence-informed foundation bridges the research-practice interface	• Achieved through literature reviews, environmental scans, review of existing competency frameworks, and expert consultation to gather evidence and contextual information • Increases validity and reliability of competency statements/ frameworks
Multi-step consensus-building process centres the process in evidence	• Expert consultation should be woven into multiple different steps of the process • Experts should include public health researchers and practitioners, as well as other actors including community-based and government partners • Centers the process in expert knowledge, research findings, and intersectoral/community-based public health
Transparent and comprehensive reporting of the methods and results for approaches is needed	• Allows for research to be interpreted and repeated • Future research should include developing validated instruments for use during consensus-building and competency theory • Pilot testing instruments before consensus-building can increase validity of results in absence of validated instruments • Reporting of methods should include participant recruitment information, detailed outline of all steps included, instruments used, and statistical methods • Supplementary information with survey instrument, interview guide, etc. would be helpful in guiding similar research and interpretation of results • Reporting of results should be systematic and detailed
Governance and coordination of competency frameworks are essential for impact	• Governance is necessary to ensure frameworks are up-to-date, resourced, and reflective of current values and needs • Coordinated and comprehensive rollout strategy impacts the success
Design of the frameworks is a key consideration for useability in practice	• Use clear language, tables, and a user-friendly design that has a reasonable number of competency statements • Future research should include an evaluation of the usefulness of the design
Multifaceted approaches for developing and evaluating competencies can address complex public health challenges	• Complex challenges must be explicitly integrated and reflected in competency frameworks • Values are key aspects of competency frameworks to address complex problems

A strong evidence-informed foundation for developing competency statements bridges the research-practice interface. The quality of the evidence used to generate findings is key to the reliability and validity of the resulting competency frameworks [47, 48]. Literature reviews, expert consultations, review of existing competency frameworks, and environmental scans can be used as a first step to gather evidence [48, 49] that can close the gap between research and practical knowledge and provides contextual evidence about the public health system in Canada. Within healthcare, a similar process is used where literature reviews [50], surveys [51], and interviews [52] were used to develop the initial list of competency statements for further consensus-building [49]. This approach is consistent with the steps outlined in the Delphi and modified Delphi techniques [48, 49].

Multi-step processes for consensus-building, including expert consultation and iterative methods, increase the validity of results through the incorporation expert knowledge and research findings [49]. Expert consultation is a key step in the consensus-building process to ensure a strong basis in the evidence [47, 48]. Practitioners and researchers are most often included in consensus-building processes in the included public health literature and within healthcare [50–52] and veterinary medicine [53, 54]. Consensus-building processes to develop competency frameworks in the included literature were most often academia-led and conducted via expert consultations, surveys, and modified Delphi technique with public health academics and practitioners. However, public health goes beyond the public health sector and the consensus-building process should include community-based organizations, government, and other stakeholders to ensure community needs and values are being met [45]. The inclusion of other actors ensures competencies are reflective of collaborative, intersectoral, community-based public action.

Transparent and comprehensive reporting of the methods and results of consensus-building processes for public health competencies and frameworks is needed so that the research can be repeated, or similar processes can be implemented, and understood. The details of the consensus-building process were lacking in some studies – a similar trend has found for competency framework development in the healthcare [50–52] and veterinary medicine [53, 54]. This is also true across health research in general where lack of transparent reporting makes interpreting the methods, evaluating the reliability and validity of results, and comparing to the wider body of knowledge difficult [55]. Research with low transparency has implications for use including possible harms and unintended consequences and reducing the possibility of benefits, contributing to lower overall public health impact [56]. Competency theory and validated instruments that guide consensus-building and ensure reliable and accurate results were identified as lacking by included studies. A threshold for agreement has also not been validated, making it difficult for studies to select an *a priori* level of agreement [34, 39]. The focus on validated instruments within competency framework research tends to be for measuring perceived competence development in education [57] or professional development [58] rather than the process itself of developing consensus when building competency frameworks. Pilot testing of instruments for measuring agreement during consensus-building can help to ensure validity before the process begins. The CONFRED-HP (COmpeteNcy FramEwork Development in health professions) recommendations for reporting provides researchers with clear descriptions of vital areas for reporting the development of competency frameworks to increase the transparency and trustworthiness of the research and allow for informed decision-making around its use [59]. The EQUATOR (Enhancing the Quality and Transparency Of health Research) Network guidelines can be used for increasing the transparency and trustworthiness of research on building competencies in public health students and practitioners [55]. Reporting guidelines are important tools that support best practices in research reporting, contributing to increased transparency and research impact [56].

Public health effectiveness relies on how practitioners combine their individual values, knowledge, skills, and behaviours to address community needs. The effectiveness of a public health team and workforce results from this combination across individuals, levels, and disciplines [60], although interdisciplinary teamwork is difficult [61]. Foundational competencies lay the groundwork for a common understanding of what is required to do good work in public health. Discipline-specific competencies allow for public health practitioners to have opportunities to learn and build on foundational competencies for additional mastery [62]. Despite this, diversity of competency frameworks and levels of competencies (expert and foundational vs. specialized) are key challenges in developing competency frameworks [63]. The diversity in competencies and the subsequent difficulties have implications for workforce planning and development and education [63]. Frameworks that combine different ways of knowing, including theory and practice-based knowledge, and practical examples can help contextualize the competencies [63] to different expertise levels and specializations within public health. Competencies related to communication and interdisciplinarity can assist with the challenges of multiple levels of expertise and foundational and discipline-specific competencies as well [25, 60, 64].

Integrating competency frameworks into practice requires governance and coordinated efforts, as identified by the included literature. Governance of public health core competencies is necessary to ensure competency frameworks are up-to-date, appropriately resourced, and reflective of current public health needs and values. Within a governance structure, funding, standards and quality assurance, and a comprehensive framework rollout plan were also identified as necessary. A lack of resources and infrastructure to support the development, implementation, evaluation, and ongoing refresh of competencies are key contextual factors influencing the success of competency frameworks [46]. This complements findings from another review indicating that a comprehensive and coordinated implementation strategy, including resources, governance, and collaboration, is associated with the use and impact of competency frameworks [25].

Design and readability of competency frameworks impacts their use and effectiveness. Competency statements should be written in clear language and be reasonable in number, and frameworks should be user-friendly, use tables, and be translated where applicable [25, 65–67]. Although there were a range of presentations in the included literature, most did not evaluate the usefulness of the design, with the exception of Shickle et al. [41], which found clear language was preferred.

Finally, a multifaceted approach to developing and measuring competencies is needed to address complex public health challenges. Complex public health issues including climate change, global health, health inequities, and racism/discrimination must be addressed, reflected, and integrated into public health competencies. Values that guide our research, practice, and community interactions must also be reflected in competency statements and frameworks to address these complex issues. Values are most often geared at individual practitioners but should also be extended to organizational values, as these create a culture and context for individual values to guide practice [68]. In the context of Indigenous health and reconciliation, it is imperative to explicitly integrate decolonization, anti-racist, and culturally appropriate public health practice into the public health competencies [36]. Within other disciplines, including midwifery [69] and healthcare [70], core competencies have been proposed to guide providers working to address health disparities and ensure culturally safe and effective services for Indigenous Peoples. Structural factors, including power dynamics, current and historical relationships, and needs and resources of marginalized groups must be incorporated into competency frameworks and directly influence use [25, 46]. Competency frameworks should be flexible enough to serve as practice guidelines that can be adapted to suit the specific context, complexity, and population requirements in which it is being applied [25].

### Limitations

Within the current study, the results could be limited by the selection of databases; however, we used a number of diverse databases and supplemented the search by hand searching and grey literature. Further, our research intended to focus on health communication, but found the literature to be greatly lacking, rendering a more focused review impossible. The inclusion of health communication as a key concept within the search strategy may have limited the results to those public health competency frameworks that included communication. Communication is identified across many disciplines, including public health, as a key competency, so this aspect may not have had a great impact on the results. The results may also be limited by language bias as studies were only included if they were published in English. Although we had no date or geographic restrictions, this language bias may have limited articles globally, especially those that are low or low-middle income countries, impacting the overall generalizability of results.

Biases created by study design of the included articles also result in limitations, including participant recruitment and the methods used to understand their perspectives. Information bias was present as most studies employ purposive sampling to select consensus-building groups [34, 35, 37, 41, 44]. There is also no established target number of participants for consensus-building processes for developing competency frameworks (Coleman et al., 2013) and sample sizes can be small [35, 41, 42]. The nature of participant experience and breadth of perspective could also be limited by the sampling [34, 37, 39, 41, 42, 44]. Further, many studies relied on expert knowledge and the literature to develop competency frameworks, but many areas of public health are rapidly expanding and changing [34] and/or lack the theoretical underpinning necessary to ensure reliable and valid results [34, 39, 41]. Some studies reported an *a priori* cut-off point for agreement [34, 39]; however, this threshold is not validated in the research [34, 39].

### Future research directions

Research is needed to distinguish between competencies required for core public health roles versus those for specialized roles. Research specific to the various disciplines, including public health communication, is needed to determine best practices for developing, implementing, and evaluating competency frameworks. Research reporting on the implementation and evaluation of foundational and discipline-specific competency frameworks is needed to help understand best practices and challenges in harmonizing varying competency frameworks in practice. The creation and implementation, reliability, and validity of a competency assessment instrument should also be explored. Some areas of literature related to competency statements and frameworks, such as health literacy, are heavily skewed towards healthcare and medicine, and more work is needed centring different topics within public health so that the focus is at the population level rather than the patient/individual level. Research to strengthen the connection between competency frameworks and the learning outcomes of public health graduate training and professional development would be valuable. Finally, research is needed to evaluate consensus-building processes from the perspective of participants and the utility and impact of the final framework, including design considerations. Literature in this area is largely focused on competency framework development and not on the evaluation of the process of development, implementation, use, or impact of the frameworks in practice.

## Conclusion

The review scopes and analyzes the literature on developing competency statements and frameworks for public health. The findings highlight the similarities in approaches for developing competency statements and frameworks across studies, including using a multi-step process that involves literature reviews, expert consultations, and consensus-building. Foundational and discipline-specific competency frameworks, as well those that are role- and expertise-specific, including for students, new practitioners, and leaders, are needed. Variation in transparency of reporting the process of developing competency statements and frameworks exists, with some studies including very detailed methods and results while others only high-level overviews. High transparency and multi-step processes are necessary to ensure the validity, reliability, and utility of competency statements and frameworks. Governance and comprehensive plans for implementation and renewal are necessary to ensure integration of competencies into professional standards and professional development. Values that reflect culture and social justice when addressing complex public health needs must be integrated into public health competency statements and frameworks.

### Electronic supplementary material

Below is the link to the electronic supplementary material.

Supplementary Material 1

## Data Availability

The datasets used and/or analysed during the current study are available from the corresponding author on reasonable request.
