# Supplementary material for: Developing public health competency statements and frameworks: a scoping review and thematic analysis of approaches
Source: BMC Public Health. 2023 Nov 13;23:2240. doi: 10.1186/s12889-023-17182-6 (PMC10644570; doi:10.1186/s12889-023-17182-6)
Supplement: Supplementary file 1 — Supplementary Material 1 [file 12889_2023_17182_MOESM1_ESM.pdf]

Supplementary Table 1: Summary of Data Extraction Results for Included Literature (n=13)

Title	Author (Year)	Document Type-Journal article, dissertation, government report	Country of Origin	Study Design	Study Aims, Purpose, Objectives	Type of Public Health Student or Practitioner	Methodology	Theories/Frameworks used	Pedagogy identified	Institution Administering Professional Development or Training	Purpose of Competency Work (e.g., building framework, developing competency in practitioner)	Existing Competency Framework Used	Competency Assessment Method	Focus of Training or Professional Development	Format of Training or Professional Development	Recommendations for Improved Competency Development	Collaborations Identified	Communication Focused (Y/N)	Bias Identified	Gaps/Limitations	Future Research Directions
Lifelong learning for public health practice education: a model curriculum for bioterrorism and emergency readiness.	Olson, D, Hoeppner, M, Larson, S, Ehrenberg, A, Leitheiser, A T (2008)	Journal Article	USA	Qualitative	Describing how the University of Minnesota School of Public Health developed and implemented a tool for lifelong learning model for bioterrorism and emergency readiness	MPH students	Conduct needs assesment for a framework to develop bioterrorism and emergency preparedness including the potential competency indicators, obtained through modified delphi-process, focus groups, lit searches, surveys. Identify learning opportunities for developing	The Benner Model of Expert Practice and Dreyfus Model	n/a	University of Minnesota	student development/building list	Cross referenced with the CDC bioterrorism and emergency readiness competencies	Among students, evaluation for the courses in preparedness, response, and recovery via course evaluations and six- and 12-month follow-up studies sponsored through UMNCPHP. They have also conducted online simulations to measure the performance of professional health workers who participated in the course and see if the curriciulum was effective in building their competencies.	Bioterrorism and emergency preparedness	MPH course	n/a	Collaboration with experts and PH professionals to develop the list	no	n/a	n/a	A need to advance competencies of the public health workforce, including professional training and distance-learning opportunities.
Identifying Core Competencies for Public Health Epidemiologists	Bondy, S J, Johnson, I, Cole, D C, Bercovitz, K (2008)	Journal article	Canada	Mixed-methods	Seeking perspectives on important competencies among epidemiologists familiar with or practicing in public health settings (local to national).	Epidemiologists	key informant interviews with 12 public health practitioners familiar with front-line epidemiologists' practice, followed by a web-based survey of members of a provincial association of public health epidemiologists (90 respondents of 155 eligible) and a consensus workshop.	n/a	n/a	Academia	Building lists	n/a	n/a	Epidemiology	n/a	n/a	Association of Public Health Epidemiologists of Ontario (APHEO)	no	Selection and information biases exists.	Key informants are "persons whose position or experience should give them some perspective on the magnitude and distribution of the problem,"16 but their identification by APHEO working group members may have limited the breadth and nature of perspectives tapped	Specific language is likely to be shaped by later discussion involving APHEO and other provincial and national COMPETENCIES FOR EPIDEMIOLOGISTS 250 REVUE CANADIENNE DE SANTÉ PUBLIQUE VOLUME 99, NO. 4 Figure 1. Overall* mapping of competency domains

The UK Public Health Skills and Career Framework - could it help to make public health the business of every workforce?	Wright, J, Mala, Rao, Walker, K (2008)	Journal article	United Kingdom	qualitative	to describe how the UK Public Health Skills and Career Framework was developed, and to invite discussion on its potential usefulness as a tool for facilitating a shared approach to strengthening public health competence within and across countries.	Public health professionals	The development of the Framework was led by the Department of Health in England; Skills for Health, the UK Skills Council with responsibility for the health sector; and the Public Health Resource Unit in Oxford, an organization offering specialist support to public health	UK Public Health Skills and Career Framework	n/a	public health	building competency framework	n/a	n/a	General	n/a		The development of the Framework was led by the Department of Health in England; Skills for Health, the UK Skills Council with responsibility for the health sector; and the Public Health Resource Unit in Oxford; Colleagues in Scotland, Wales and Northern Ireland	no	no	n/a	The UK team would welcome feedback on the Framework, and would also be interested to hear from colleagues in other countries who would be interested in piloting these competences for their relevance and feasibility amongst both their local public health and wider
Constructing a general competency model for Chinese public health physicians: a qualitative and quantitative study.	Shi, Lei, Fan, Lihua, Xiao, Hai, Chen, Zhenkang, Tong, Xinfa, Liu, Ming, Cao, Depin, Lei, Shi, LiHua, Fan, Hai, Xiao, ZhenKang, Chen, XinFa, Tong, Ming, Liu, DePin, Cao (2019)	Journal article	China	Mixed-methods	to construct a general model of the competencies required by Chinese public health physicians. This study is intended to lay the foundation for promoting reform of public health education in China, and may help perfect the testing and grading system of public health courses	Public Health physicians	The behavioral event interviews were used to collect data on knowledge, skills and performance characteristics of public health physicians for coping with public health events. A random stratified sampling survey was used to select public health professionals and workers	n/a	n/a	Academia	Building lists	Scanned lists from Canada, USA, UK, Australia to develop theirs	n/a	General	n/a	n/a	n/a	no	Recall bias	Chinese public health physicians are distributed across many institutions; the model does not consider the impact of minority cultures; this model is only applicable to the entry standard of public health physicians.	study the core competencies of each type of public health physician
Epidemiology competency development and application to training for local and regional public health practitioners.	Baseman, Janet G, Marsden-Haug, Nicola, Holt, Victoria L, Stergachis, Andy, Goldoft, Marcia, Gale, James L (2008)	Journal article	USA	qualitative	developing competency-based epidemiology training for non-epidemiologist public health practitioners in the northwestern United States.	Non-epidemiologist public health practitioners	Development of competencies, implementation in curriculum, evaluation of the programs	n/a	n/a	University of Washington	practitioner development/building list	Emergency Preparedness Core Competencies for Public Health Workers and Informatics Competencies for Public Health Professionals	post-course surveys (perceived)	Epidemiology	Online modules and in-person course		local and regional public health partners and the University	no	no	Subjective assessment of the trainings, difficult to measure impact	

Domains of Core Competency, Standards, and Quality Assurance for Building Global Capacity in Health Promotion: The Galway Consensus Conference Statement	Allegrante, J P, Barry, M M, Airhihenbuwa, C O, Auld, M E, Collins, J L, Lamarre, M C, Magnusson, G, McQueen, D V, Mittelmark, M B (2009)	Conference Report	Ireland	qualitative	The paper reports the outcome of the Galway Consensus Conference, an effort undertaken as a first step toward international collaboration on credentialing in health promotion and health education	Health promotion practitioners	Twenty-nine leading authorities in health promotion, health education, and public health convened a 2-day meeting in Galway, Ireland, during which the available evidence on credentialing in health promotion was reviewed and discussed. Conference participants reached	n/a	n/a	n/a	Building lists	n/a	n/a	Health promotion	n/a	standards and quality assurance mechanisms need to be in place at training institutions wherever in the world they are located. Every relevant training authority should strive to develop quality assurance mechanisms appropriate to the prevailing political, economic, and cultural circumstances. Where a train-the-trainer model is employed at the level of practice, the trainers should have formal preparation in health promotion and demonstrate proficiency in the domains of core	29 leading authorities in health promotion and education	no	no	n/a	The Galway Consensus Conference participants urge all relevant institutions and all interested individuals to use meetings, conferences, journals, and Internet resources to continue a lively dialogue on these important issues
Identifying core competencies for practicing public health professionals: results from a Delphi exercise in Uttar Pradesh, India	Bhandari, Wahl, Bennett, Engineer, Pandey, & Peters (2020)	Journal article	India	Mixed methods	This study aims to identify the requisite core competencies for practicing health professionals in mid-level supervisory and program management roles to effectively perform their public health responsibilities in the resource-poor setting of Uttar Pradesh (UP), India.	List for practicing public health professionals in mid-level supervisory and program management roles	Narrative review of core competency frameworks, key informant interviews to prepare a list of initial competency statements. Day-long workshop with 22 ph eexperts and government officials to revise the list. Revised list rated and then aggregate scores discussed by	multi-step interactive Delphi technique called Estimate-Talk-Feedback-Estimate (EFTE)	n/a	State Institute of Health and Family Welfare provided technical support. Johns Hopkins Bloomber School of Public Health-lead author and delphi technique facilitation. Bill and Melinda Gates Foundation funded the project. A diverse group of 22 participants with backgrounds in public health, professional education, and human resources for health representing the Government of UP (GOUP), academia, and public health Non-Governmental Organizations (NGOs) in India participated	Develop a list of agreed upon competencies for public health professionals in leadership roles in India	core competency frameworks from Canada, Europe, New Zealand, Spain, United Kingdom, United States, and the Americas	n/a	General	n/a	The onus then falls on the user—policymaker or educator—of the framework to understand the importance of individual core competency to a specific position as they may vary depending on the position. The user should evaluate the types of positions and career trajectories when planning competency-based professional development to ensure that an organization collectively has the strengths across these competencies The second way to	Collaboration between a university and government agency in India to develop list of competencies	Communication was one of 8 overall domains of competencies developed with 5 subcompetencies identified.	Regarding the composition of the expert panel, panelists for this study were chosen after extensive consultations and online searches. However, some potential participants declined the invitation due to a lack of availability. These non-participants were similar in their backgrounds from the participants in the Delphi process. The EFTE technique used in this study required	We identified an important theoretical gap related to the core competency development process, which is relevant for researchers in UP and elsewhere. Core competency frameworks need to be broad enough to be comprehensive but also targeted enough to be relevant	Future research can assess the reliability and validity of that instrument, which can then be used to evaluate levels of competencies of health professionals working in public health management and supervisory roles.

The applicability of the UK Public Health Skills and Knowledge Framework to the practitioner workforce: lessons for competency framework development	Shickle, Stroud, Day, & Smith (2018)	Journal article	United Kingdom- England, Scotland, Wales and Northern Ireland	Qualitative	One of the priorities of the UK Faculty of Public Health Workforce Strategy & Standards Document 2018–218 was to define 'standards for the necessary professional workforce required to enable transformations in health and wellbeing of the population to take place' including 'the development of an effective public health practitioner workforce'. The initial objective of	Public health practitioners within 8 local health authorities	15 small group interviews involving 51 participants were conducted across the 8 health authorities. The interviews also covered appropriateness of the PHSKF as a basis for degree curricula and apprenticeship schemes.Interviews were recorded and transcribed. A	The UK Public Health Skills and Knowledge Framework (PHSKF)	n/a	University of Leeds	To assess the extent to which practitioners utilize competencies defined within the UK Public Health Skills and Knowledge Framework (PHSKF).	The UK Public Health Skills and Knowledge Framework (PHSKF)1 has 70 competencies categorized within 13 functions	Small group interviews were used to assess if particpants had seen and used the PHSKF.		It will be important to assess whether and how the updated PHSKF is being used, otherwise the utility of such frameworks is brought into question. Group participants were supportive of apprenticeships and a prospective training programme that allowed apprentices to rotate between training opportunities so that they could develop proficiency and document experience for all of the PHSKF competencies. Some participants with less senior	Not identified- authors from University of Leeds	Communicate with others to improve health outcomes and reduce health inequalities There was widespread recognition of the importance of communication across the range of individuals and organizations. There was also a recognition of the need to coordinate communications to prevent duplication both within own and with other organizations. Some practitioners were using the	The vast majority of mid-level public health staff do not have 'practitioner' in their job title, and the lack of an agreed definition meant that it was left to recipients of the invitation letter to decide who was suitable to be interviewed. It would also have been desirable to analyse the	Future research should apply a more explicit definition of practitioner in order to assess the skills and knowledge of specific sections of the public health workforce.It was also difficult to assess specific individual's competencies. Instead there was a tendency to assess the
Comptency-to-Curriculum Toolkit	Gebbie (2008)	Grey Literature	USA	N/A- toolkit	The Competency-to-Curriculum Toolkit is presented to the public health workforce training and education community as an aid in assuring that the workforce, key to the public health infrastructure, is truly competent to perform essential public health services in all areas of public health practice.	General public health practitioner to move from a competency set to curriculum for public ehalth practitioners and students	The steps to move from competencies to curricula in this description are: • Specify the audience, • Develop learning objectives, • Assess the time availability of the learner, • Determine how and when learning will be measured, • Determine expected outcomes,			Academia	Building lists (pg 13) and matching to curriculum development	A Collection of Competency Sets of Public Health-Related Occupations and Professions		General			As the practice field evolves, for example, competencies in communication for use in informing communities and mobilizing partnerships (two essential public health services) should be reviewed to assure that they are necessary, accurate and timely.		

Review of Core Competencies for Public Health: An Aboriginal Public Health Perspective	Hunt (2015)	Grey Literature	Canada	Qualitative	As can quickly be determined by looking at the competency sets, they frequently intersect or overlap. In some cases, the same general area of practice or performance has been addressed in several competency sets. While it is hoped that increased communication among those interested in workforce development will decrease the development of what appear to be	As can quickly be determined by looking at the competency sets, they frequently intersect or overlap. In some cases, the same general area of practice or performance has been addressed in several competency sets. While it is hoped that increased communication among those interested in workforce development will decrease the development of what appear to be duplicative sets, there will still be times when an individual or an organization	Review of Core Competencies for Public Health in Canada 1.0 from an Aboriginal public health perspective, building on current international work on the public health of Indigenous peoples. The document has been peer reviewed by experts.		NCCAH	Adapting the PHAC competencies for Indigenous health	PHAC Competency Framework		NCCAH	Better recognize the interrelatedness of cultural safety, communication, and relationship-building. Include a comptenecy on the ability to work effectively across language barriers.			In light of recent iniatitives in Aboriginal public health, it is timely to update the 2007 Core Competencies for Public Health in Canada 1.0 in order to make them relevant to healthcare provision for First Nations, Inuit and Metis people.
Health Literacy Practices and Educational Competencies for Health Professionals: A Consensus Study	Coleman, Hudson, & Maine (2013)	Journal article	USA	Mixed methods	This study aimed to identify a set of health literacy educational competencies and target behaviors, or practices, relevant to the training of all health care professionals.	General public health- issue affects all disciplines within ph	The authors conducted a thorough literature review to identify a comprehensive list of potential health literacy competencies and practices, which they categorized into 1 or more educational domains (i.e., knowledge, skills, attitudes) or a practice domain. The authors stated each	Bloom's Taxonomy of Educational Objectives used to operationalize competencies	Academia	Building list for health literacy competency	No- literature search used to identify items within knowledge, skills, attitidues, practices	Health Literacy		Our study focused on health literacy competencies and practices to the extent that health literacy can be separated from other health communication constructs. Despite some obvious areas of overlap, no specific attempt has been made to systematically integrate the health literacy competencies and practices identified here with other essential elements of communication competency,	First, although the list of potential competencies and practices identified by the nonsystematic literature review was extensive, it may not have been exhaustive and may reflect the unconscious biases of the compiling author (C.C.). The boundaries between health literacy and other health communication constructs (e.g., crosscultural communicatio	Most health literacy recommendations found in the literature are based on expert opinion, and, with a few exceptions, lack rigorously established supporting evidence (Sheridan et al., 2011).Last, and perhaps most important, although it may be tempting to rank-order	However, additional work is now needed to help prioritize these competencies and practices, particularly in identifying those with the greatest potential effect on outcomes for patients. In their current state, the lists of competencies and practices identified in this study are

Competencies for Health Communication Specialists: Survey of Health Communication Educators and Practitioners	Park, Harrington, Crosswell, & Parvanta (2021)	Journal article	USA	Quantitative	This research picked up where previous work left off by developing a systematic, comprehensive list of competencies to be broadly considered for graduate programs offering health communication degrees.	graduate students in health communication	To prepare the survey, a working group drafted a competency list through reviewing the literature and soliciting feedback from attendees at a professional society meeting, resulting in lists of items reflecting 18 knowledge domains, 11 skill sets, and 14 application	KSA (knowledge skills abilities) model		Academia	Building list	No- literature search used to identify competencies		Health communication	Yes	First, the sample size is rather small and not representative of health communication educators and practitioners in the United States.Second, the attrition rate was high between survey initiation and the questions concerning knowledge domains, which is understandable given	We hope that lessons from this pandemic and the social unrest stay with us long after humanity finds its way out of the current crisis and that health communication competencies guide academic programs to design their curricula to address these two pressing public health
Developing Competencies for a Graduate School Curriculum in International Health	Hagopian, Spigner, Gorstein, Mercer, Pfeiffer, Frey, Benjamin, & Gloyd (2008)	Journal article	USA	Mixed methods	The purpose of this article is to report on how we developed international health competencies to guide our curriculum development.	MPH with speciality in global health	Began with literature search to find a published set of competencies that could be modify and adapted for the program. Nothing existed so used US Council on Linakes US competencies list. Department also developed curriculum competencies for the generic MPH program and used the UN		interdisciplinary course offerings, student portfolios, and culminating projects (such as a thesis)	Academia	Buildling list	US Council on Linkages 2001	self-rated baseline of current competencies for incoming students so they can compare after the two years in the program.			Little literature in the area. Higher response rate would make for more robust data. No previous baseline survey to compare responses to.	
